# Life-Cycle Performance of Poly(methyl methacrylate) in Digital Dentistry: A Critical Review of Material Efficiency, Waste Generation, and Circularity

**DOI:** 10.3390/polym18172071

**Published:** 2026-08-26

**Authors:** Claudia Florina Bogdan-Andreescu, Andreea-Mariana Bănățeanu, Cristina Chelu, George Ion, Vivyiana Paraschiv, Ștefan-Dimitrie Albu, Dan Alexandru Slăvescu, Manuela Victoria Chivu, Dorin Alexe, Eugenia Diana Rădulescu

**Affiliations:** 1 Department of Speciality Disciplines, Faculty of Dental Medicine, “Titu Maiorescu” University, 031593 Bucharest, Romania; claudia.andreescu@prof.utm.ro (C.F.B.-A.); andreea.banateanu@prof.utm.ro (A.-M.B.); cristina.chelu@prof.utm.ro (C.C.); manuela.chivu@prof.utm.ro (M.V.C.); dorin.alexe@prof.utm.ro (D.A.); diana.radulescu@prof.utm.ro (E.D.R.); 2 Department of Prosthetic Dentistry, Faculty of Dentistry, “Carol Davila” University of Medicine and Pharmacy, 020021 Bucharest, Romania; 3 Department of Periodontology, Faculty of Dentistry, “Carol Davila” University of Medicine and Pharmacy, 020021 Bucharest, Romania; stefan.albu@umfcd.ro; 4 Department of Dentistry, Faculty of Medicine and Pharmacy, University of Oradea, 410073 Oradea, Romania; slavescudan@uoradea.ro

**Keywords:** circular economy, depolymerization, dental polymer, digital dentistry, environmental sustainability, life-cycle assessment, monomer recovery, polymethyl methacrylate, prosthodontics, recycling

## Abstract

Poly(methyl methacrylate) (PMMA) is one of the most widely used polymeric biomaterials in prosthodontics and digital dentistry because of their clinical reliability and compatibility with computer-aided design/computer-aided manufacturing (CAD/CAM). Its widespread use raises questions regarding material consumption, manufacturing waste, recyclability, and circularity. A critical narrative review supported by a structured literature search was conducted. PubMed, Scilit, OpenAlex, and ScienceDirect were searched for English-language literature published from January 2000 to June 2026. Targeted Google Scholar searches, cross-referencing, standards, and official technical sources supplemented the search. Evidence was organized according to its directness to dental PMMA and synthesized thematically. Prepolymerized CAD/CAM PMMA provides consistent material quality and generally improved mechanical performance compared with conventionally processed PMMA; however, subtractive manufacturing generates disc remnants, milling particles, and polishing residues. Mechanical recycling and depolymerization demonstrate technical recovery potential, although evidence specific to heterogeneous dental waste streams, environmental performance, and clinical-grade reuse remains limited. Technical recyclability should not be automatically equated with a viable circular economy or a net environmental benefit. Future research should quantify dental PMMA waste, establish effective collection and recovery pathways, and integrate life-cycle assessments with clinical performance and safety standards.

## 1. Introduction

Healthcare systems consume substantial energy and raw materials and generate greenhouse gas emissions and waste, with the global healthcare sector estimated to account for approximately 4.4% of global net emissions [1,2,3]. These environmental burdens have increased the need to complement clinical effectiveness with environmental stewardship and more efficient resource management. Dentistry is part of this transition, extending sustainability considerations to the selection, processing, use, and end-of-life management of dental materials.

Dental biomaterials have traditionally been evaluated primarily in terms of mechanical performance, esthetics, biocompatibility, and clinical durability [4,5,6]. However, the expansion of computer-aided design and computer-aided manufacturing (CAD/CAM), intraoral scanning, additive manufacturing, and artificial intelligence (AI) has transformed dental workflows while also changing patterns of energy use, material consumption, and waste generation [7,8,9,10]. Consequently, the assessment of digital dentistry should extend beyond clinical and technical performance to include environmental effects across the material life cycle [7,11,12].

Poly(methyl methacrylate) (PMMA) is particularly relevant to this evaluation because it combines a long clinical history with extensive use in conventional and digital prosthodontics [5,6,13,14,15,16,17]. Despite the increasing availability of ceramics, composite resins, high-performance polymers, and reinforced materials, PMMA remains a reference polymer for removable and provisional applications because of its processability, esthetic properties, affordability, and generally reliable clinical performance [5,15,18,19,20,21]. Its continued use therefore makes the material flows and waste associated with its manufacture increasingly relevant to sustainability assessment.

Digital manufacturing has reinforced the role of PMMA in prosthodontics while altering its material flows. Industrially polymerized CAD/CAM blanks provide more homogeneous and consistent material than many conventionally processed acrylic resins, while digital records support standardized fabrication and reproducible replacement [7,15,22]. However, subtractive fabrication inherently generates unused blank material and machining residues [7,23,24]. Although mass- and item-based dental waste audits demonstrate that material flows can be quantified systematically, comparable standardized assessments of PMMA-disc utilization and waste generation remain lacking [25].

The environmental implications of PMMA extend beyond the dental laboratory. Commercial PMMA is predominantly produced from petrochemical feedstocks, is not biodegradable under environmental conditions, and involves energy- and resource-intensive monomer synthesis and polymer manufacture [26,27,28,29]. Its environmental performance therefore depends on impacts across the life cycle, from raw-material and MMA production to fabrication, clinical use, and end-of-life management. Life-cycle assessment (LCA), standardized by ISO 14040 and ISO 14044, provides a framework for evaluating these interconnected stages [30,31] and comparing materials and production pathways [32]. However, its application to dental biomaterials and prosthetic workflows remains limited [33,34], while broader approaches to resource efficiency, eco-design, sustainable procurement, and circular economy have only recently gained attention in dental materials research [32,33,35].

PMMA is of particular interest because its polymer chemistry permits several recovery pathways. Clean thermoplastic residues may be mechanically reprocessed, while chemical or thermal depolymerization can recover MMA for subsequent purification and repolymerization [27,28,36,37,38,39,40,41]. Although the broader polymer literature establishes the technical recyclability of suitable PMMA streams, recent dental studies provide more limited proof-of-concept evidence, including depolymerization of PMMA-based dental waste and reincorporation of particles recovered from milled CAD/CAM discs into denture-base materials [23,42]. However, closed-loop feasibility depends on feedstock homogeneity, source segregation, contamination control, and the properties of the recovered material [43]. Thus, dental PMMA waste may retain recoverable material value, but its routine reintegration into a validated circular system has not yet been demonstrated.

Technical recyclability, however, should not be equated with practical circularity or demonstrated environmental benefit. Mechanical recycling requires sufficiently clean and compositionally compatible feedstock, whereas depolymerization depends on collection volumes, contamination control, process efficiency, energy demand, monomer purification, and viable reintegration of the recovered material [27,28,38,39,40,41]. These requirements are particularly relevant to heterogeneous dental PMMA waste streams, which range from unused disc remnants and milling particles to polishing dust and devices that have entered clinical use. Additives, cross-linking agents, processing fluids, biological contamination, and mixing with other resin-based materials may further constrain recovery. Therefore, recycling should be evaluated at the system level and compared with waste prevention, improved CAD nesting, extended service life, and other strategies for reducing virgin-material demand [30,31,32].

This distinction highlights an important implementation gap. Despite the availability of PMMA recovery technologies, dental residues from milling and laboratory processing largely remain within a linear production–use–disposal model [7,27,41]. Achieving circularity requires not only technical recoverability but also source segregation, traceability, safe handling, collection infrastructure, quality assurance for recovered materials, and viable economic and regulatory conditions [32,41]. Dental PMMA therefore provides a relevant case for examining the gap between technical recyclability and implementation within a circular healthcare system.

The literature remains fragmented across dental materials science, digital manufacturing, polymer recycling, environmental assessment, and waste management. Most dental PMMA studies focus on mechanical and clinical performance, including strength, fracture behavior, wear, surface properties, bonding, and manufacturing accuracy [5,6,15,22], whereas relatively few quantify PMMA waste generation, evaluate recovery processes, or assess environmental outcomes in dental settings [23,42]. Conversely, the broader polymer-recycling literature provides substantial evidence on PMMA recovery but does not fully address the composition, contamination, scale, regulatory requirements, and clinical constraints of dental waste streams [27,28,36,37,38,39,40,41]. A critical synthesis is therefore needed to connect material performance with waste generation, recovery, and life-cycle impacts while distinguishing direct dental evidence from evidence transferred from other PMMA applications.

Accordingly, this review critically examines PMMA in digital dentistry through four connected perspectives: material and clinical performance; waste generation during digital manufacturing; mechanical and chemical recovery pathways; and the conditions required to translate technical recyclability into measurable circularity and environmental benefit. It further evaluates the available life-cycle evidence and proposes a conceptual sustainability framework and a practical waste-management protocol for future validation in digital dental laboratories. By integrating these domains, this review aims to connect material performance with life-cycle and circular-economy decision-making.

The contribution of this review lies in integrating domains that have largely been considered separately in the dental literature. Rather than treating PMMA primarily as a clinical biomaterial or considering recycling as an isolated end-of-life option, this review links material and clinical performance with digital-manufacturing material flows, waste characteristics, recovery pathways, LCA, and the organizational and regulatory conditions required for circular implementation. A further contribution is the explicit separation of direct dental-PMMA evidence from adjacent dental evidence, indirect polymer evidence, and broader contextual evidence, allowing the limits of extrapolation to dental practice to remain visible. On this basis, this review develops a conceptual sustainability assessment framework and identifies measurable priorities for future validation rather than proposing a clinically validated circularity model.

## 2. Materials and Methods

### 2.1. Review Design

This article was designed as a critical narrative review supported by a structured literature search. The approach was selected because the research question covers dental materials science, digital manufacturing, polymer recycling, life-cycle assessment (LCA), waste management, and circular-economy implementation. These domains contain heterogeneous evidence, including laboratory studies, LCA studies, narrative and systematic reviews, standards, technical reports, and official documents that cannot be meaningfully combined through meta-analysis.

This review was informed by established principles of integrative and narrative evidence synthesis [44,45]. A structured literature search and explicit description of source selection were used to improve transparency and traceability. Selected PRISMA 2020 reporting principles were used solely to improve transparency in documenting literature identification and study selection [46]. Accordingly, this article should be interpreted as a critical narrative review supported by a structured search rather than as a systematic review.

This review addressed the following connected questions: which material and processing characteristics influence the performance and recoverability of dental PMMA; which waste streams are generated during digital dental manufacturing; which mechanical and chemical recovery pathways may be applicable to these streams; and which environmental, organizational, quality, and regulatory conditions are required to translate technical recyclability into practical circularity.

### 2.2. Information Sources and Search Strategy

Structured searches were conducted in PubMed, Scilit, OpenAlex, and ScienceDirect. PubMed was used primarily to identify dental and biomedical literature; Scilit and OpenAlex broadened retrieval across dentistry, materials science, polymer engineering, and environmental research; and ScienceDirect was searched for relevant literature on digital manufacturing, polymer processing, waste, recycling, and environmental performance (Table 1).

The PubMed, Scilit, OpenAlex, and ScienceDirect searches were completed on 30 June 2026. The predefined publication period extended from 1 January 2000 to 30 June 2026, and the structured searches were restricted to English-language publications.

The reference lists of potentially relevant articles were screened manually to identify additional eligible publications. Targeted Google Scholar searches were also undertaken when necessary to locate studies addressing specific concepts that were insufficiently represented in the main database results, including dental PMMA waste generation, mechanical recycling, thermal depolymerization, methyl methacrylate recovery, life-cycle assessment, microplastic release, and bio-based or blended PMMA materials.

Standards, reports from recognized professional or governmental organizations, and official technical sources were consulted when peer-reviewed publications did not provide sufficient information on terminology, waste management, material safety, or regulatory requirements. These supplementary sources were used for contextual interpretation and were not included in the numerical database-screening flow.

### 2.3. Search Strategies

The search strategy combined terms related to PMMA, dental and digital-manufacturing applications, and sustainability or end-of-life management. The core concepts included “PMMA” or “polymethyl methacrylate” combined with terms related to digital dentistry, CAD/CAM, milling, additive manufacturing, dentures, and prosthodontics, together with recycling, waste, depolymerization, environmental impact, and circular economy. The strategy was adapted to the search fields and Boolean functions available in each database. Because ScienceDirect did not reliably process the complete search string in a single query, three shorter searches were conducted using its advanced-search fields. Complete database-specific search strategies, search fields, and numbers of records retrieved are provided in Supplementary Table S1.

### 2.4. Eligibility Criteria

Publications were considered eligible when they addressed at least one of the following:

- PMMA materials used in dentistry, prosthodontics, or digital dental manufacturing;
- Subtractive or additive manufacturing of PMMA-based dental devices;
- Generation, characterization, collection, or valorization of dental PMMA waste;
- Mechanical recycling, dissolution, thermal or catalytic depolymerization, or methyl methacrylate recovery from PMMA;
- Reuse of recovered PMMA or methyl methacrylate in dental or non-dental applications;
- Environmental impacts, LCA, material efficiency, microplastic release, or circular-economy considerations relevant to PMMA;
- Development of bio-based, blended, reinforced, or otherwise modified PMMA materials with potential sustainability implications.

Original research articles, review articles, and relevant technical or methodological studies published in English were eligible. Evidence from outside dentistry was retained only when it directly informed PMMA recycling, depolymerization, monomer recovery, environmental performance, or circularity and when equivalent dental evidence was unavailable.

Publications were excluded when they

- Did not address PMMA or a directly relevant PMMA-containing material;
- Focused on dental materials without a meaningful connection to PMMA, material efficiency, waste, recycling, or environmental impact;
- Concerned only conventional clinical performance without relevance to digital manufacturing or sustainability;
- Addressed unrelated industrial, biomedical, optical, electronic, or construction applications without transferable relevance to dental PMMA;
- Were conference abstracts, editorials, letters, news items, or other records that did not provide sufficient methodological or technical information;
- Were not available in English;
- Fell outside the predefined publication period; or
- Duplicated another retrieved record.

No study was excluded solely because its results were negative, inconclusive, or unfavorable to recycling or sustainability.

### 2.5. Record Management and Study Selection

Records retrieved from PubMed, Scilit, and OpenAlex were imported into Rayyan for duplicate identification and title-and-abstract screening. PubMed yielded 650 records, Scilit yielded 21 records, and OpenAlex yielded 287 records, producing 958 imported records.

The ScienceDirect searches yielded five records, which were assessed directly on the ScienceDirect platform and were excluded because they did not meet the eligibility criteria.

After removal of 96 manually verified duplicates, 862 unique records underwent title-and-abstract screening according to the predefined eligibility criteria. Of these, 846 were excluded, and 16 were retained after full-text assessment for inclusion in the critical synthesis. Complementary searching, including targeted Google Scholar searches and citation chasing, identified three additional eligible studies that were not retrieved through the structured database screening. Consequently, 19 studies were included in the final critical synthesis (Table 2). The literature-identification and study-selection process is summarized in Supplementary Figure S1.

### 2.6. Data Charting

For each publication retained from the structured search, the following information was charted, when available:

- Authors and year of publication;
- Study design and application field;
- PMMA source, formulation, or manufacturing route;
- Type of dental application or waste stream;
- Recycling, recovery, or valorization process;
- Proportion of recycled material or recovered monomer;
- Reported mechanical, physical, chemical, biological, or environmental outcomes;
- Presence of quantitative material-flow or life-cycle data;
- Principal findings and limitations; and
- Directness of the evidence to dental PMMA.

Because the studies differed substantially in their objectives, materials, experimental conditions, outcome measures, and reporting practices, the results were synthesized narratively rather than statistically.

### 2.7. Evidence Classification and Critical Appraisal

The evidence was classified according to its directness to the review question:

- Direct dental evidence: studies involving dental PMMA, dental devices, dental manufacturing processes, or dental PMMA waste;
- Adjacent dental evidence: studies involving other dental polymers or dental manufacturing waste with clearly transferable implications for PMMA management;
- Indirect polymer evidence: studies of non-dental PMMA recycling, depolymerization, monomer recovery, or environmental behavior used to assess technical feasibility;
- Contextual evidence: standards, life-cycle frameworks, policy documents, technical reports, and broader sustainability literature.

Greater interpretive weight was assigned to direct dental evidence. Findings from adjacent dental or non-dental polymer studies were explicitly identified as transferable or indirect and were not presented as demonstrating the clinical feasibility of closed-loop dental PMMA recycling.

Critical appraisal focused on the relevance of the material and waste stream, experimental transparency, sample characterization, comparability with dental PMMA, reporting of recovery yield, mechanical or chemical performance, contamination control, safety considerations, and the presence of environmental or life-cycle data. Particular attention was given to whether a study demonstrated only technical recyclability or also addressed collection efficiency, traceability, recovered-material quality, clinical safety, and net environmental benefit.

### 2.8. Evidence Synthesis and Conceptual Framework

The extracted evidence was organized thematically around the following:

- PMMA composition, production, and dental applications;
- Performance of PMMA in digital dentistry;
- Material consumption and waste generation in subtractive and additive workflows;
- Mechanical recycling and incorporation of recovered polymer;
- Thermal and chemical depolymerization and methyl methacrylate recovery;
- Environmental implications, including microplastics and life-cycle considerations;
- Bio-based and modified PMMA materials; and
- Requirements for circular implementation in dentistry.

The synthesis distinguished among demonstrated findings, proof-of-concept results, and proposed future pathways. Technical recyclability was not considered equivalent to demonstrated circularity. A circular pathway was considered substantiated only when evidence addressed the broader system requirements of waste collection, separation, traceability, recovery yield, material quality, safety, clinical performance, and life-cycle benefit.

## 3. Evolution of the Evidence on PMMA in Digital Dentistry

### 3.1. From Material Performance to Material Flows

Research on dental PMMA has historically been dominated by questions of mechanical, physical, and biological performance. Early studies concentrated on flexural and fracture behavior, polymerization shrinkage, residual monomer, surface properties, color stability, wear, and biocompatibility [4,5,6,63]. This evidence established the clinical utility of PMMA but generally treated the material as a finished biomaterial rather than as part of a wider production and end-of-life system.

The adoption of CAD/CAM shifted attention toward industrially polymerized blanks, manufacturing accuracy, dimensional stability, and reproducibility [13,15]. Clinical and laboratory research subsequently expanded across provisional restorations, complete dentures, implant-supported prostheses, and other digitally fabricated devices [7,13,16,55,64,65]. This body of work supports the functional relevance of milled PMMA, but it rarely reports the mass of the original disc, the fraction incorporated into the device, or the quantity and composition of residues generated during milling. Consequently, evidence of clinical performance is substantially stronger than evidence of material efficiency.

Sustainability-oriented publications have emerged more recently, drawing on polymer recycling, LCA, and circular economy concepts [27,28,30,31]. The resulting field is therefore asymmetric: mature evidence describes how PMMA performs, whereas a much smaller and more heterogeneous evidence base addresses how it is produced, discarded, recovered, or recirculated.

### 3.2. Structure and Directness of the Available Evidence

The literature relevant to sustainable dental PMMA can be organized into four evidence categories. The first comprises direct dental-PMMA studies, including investigations of recycled denture-base PMMA, particles recovered from milled CAD/CAM discs, and depolymerization of dental PMMA residues [23,41,42,52,54,60]. The second includes adjacent dental evidence, including studies of other CAD/CAM materials, digital-workflow LCA, microplastic release, and waste-derived fillers. The third consists of transferable evidence from non-dental PMMA recycling and polymer engineering. The fourth provides contextual evidence from sustainable healthcare, waste-management, and circular-economy frameworks.

Evidence categories were not treated as interchangeable. Direct dental-PMMA studies were used preferentially to support conclusions concerning dental waste generation, recovery, and reuse, whereas non-dental PMMA and broader polymer-recycling studies were used primarily to establish technical principles and potential recovery pathways. Where conclusions rely on transferable evidence, their applicability to dental practice is explicitly qualified because differences in feedstock composition, contamination, collection scale, processing conditions, and regulatory requirements may substantially affect feasibility and environmental performance.

Reviews and bibliometric analyses indicate increasing interest in digitally manufactured materials, multifunctional modifications, and sustainability in oral healthcare [66,67,68,69,70]. Nevertheless, the dominant experimental themes remain strength, fracture toughness, surface roughness, wear, microbial adhesion, bonding, and dimensional accuracy [5,6,13,15,16,17]. Direct measurements of PMMA waste generation, recovery yield, recycled-material quality, or environmental impact remain uncommon [18,27,28,30,31]. The apparent growth of sustainability-related publications should therefore not be interpreted as a mature PMMA-specific evidence base.

Table 2 makes this distinction explicit. Only a small number of the core studies investigate recovery of dental PMMA directly. Other studies are valuable because they clarify material substitution, particulate hazards, digital-workflow impacts, or circular management of analogous dental wastes, but their conclusions cannot be transferred to PMMA without limitations. In particular, zirconia residues, thermoplastic PMMA, PMMA/PLA blends, and cross-linked printable methacrylate resins differ in composition and recovery behavior.

### 3.3. What the Current Evidence Supports

Three conclusions are reasonably supported. First, subtractive CAD/CAM fabrication generates identifiable PMMA waste streams, including unused disc material and milling residues [7,23,24,62]. Second, suitable PMMA can be recovered through mechanical reprocessing or through chemical, thermal, catalytic, and solvent-assisted routes that target polymer purification or MMA recovery [23,41,42,60,71,72,73,74,75]. Third, practical implementation depends on source segregation, contamination control, collection, and access to appropriate processing infrastructure [76,77].

The strength of support differs across these conclusions. The generation of residues is inherent to subtractive manufacturing, but dental studies seldom quantify it using consistent mass-based indicators. The technical recoverability of PMMA is supported largely by polymer-engineering studies conducted with better characterized and larger-volume feedstocks than those available in dental laboratories. Direct dental studies demonstrate proof of concept, not yet a validated closed-loop system. Accordingly, the evidence supports technical potential more strongly than routine feasibility.

LCA provides the appropriate framework for determining whether a recovery pathway produces an environmental benefit across raw-material production, manufacturing, transport, use, and end-of-life stages [30,31,78]. Healthcare case studies show that waste-management choices can materially affect environmental outcomes [79], while emerging dental assessments demonstrate the importance of functional units, system boundaries, energy sources, transportation, and replacement rates [58,61]. However, no sufficiently developed body of PMMA-specific dental LCA currently establishes that a particular recycling pathway is environmentally preferable under routine laboratory conditions.

### 3.4. Claims That Remain Unresolved

The distinction between demonstrated findings and plausible but unvalidated propositions is particularly important in this field. Current dental evidence supports the generation of identifiable PMMA waste streams and provides proof of concept for selected recovery approaches; however, evidence of technical recovery should not be interpreted as demonstration of routine closed-loop recycling, clinical-grade reuse, or net environmental benefit.

The available literature does not yet establish the quantity or composition of PMMA waste generated per dental device, the degree of contamination across different residue streams, or the proportion that can be collected economically [23,25,42,62]. Available studies provide selected mechanical, surface, and biological outcomes for recycled dental PMMA, but they do not establish whether recovered PMMA or MMA can repeatedly meet the chemical, mechanical, biological, and regulatory requirements of dental applications [23,42,52,54,60]. Biological evidence is particularly limited: available studies include selected in vitro outcomes for recycled denture-base PMMA, whereas comprehensive assessment of cytotoxicity, residual monomer and leachables, aging-dependent effects, repeated recycling, and in vivo or clinical biocompatibility remains insufficient [52,54]. Data on occupational exposure, transport distances, purification demand, recycling yield, and displacement of virgin MMA remain insufficient for environmental comparison.

For these reasons, PMMA recycling technologies should not be described as fully mature in the dental context. Industrial technical maturity does not demonstrate dental-system readiness. Likewise, recyclability is a material or process capability, whereas circularity requires verified retention of material value within an operational collection, recovery, quality-assurance, and reintegration system. Net sustainability additionally requires evidence that the circular option reduces environmental burdens rather than shifting them to energy use, transport, solvents, emissions, or unusable secondary outputs.

### 3.5. Implications for Sustainable Digital Dentistry

Contemporary healthcare frameworks increasingly define value as the integration of clinical outcomes, resource efficiency, environmental stewardship, and system resilience [3,80,81]. Circular-economy research in healthcare further shows that technological options must be connected to procurement, logistics, governance, safety, and stakeholder coordination [82]. Applied to dental PMMA, this perspective requires performance and environmental evidence to be considered together rather than treating recycling as an isolated end-of-life intervention.

The research priority is therefore not simply to identify additional recovery technologies, but to characterize dental PMMA material flows and test realistic pathways. Standardized studies should report disc utilization, waste mass and composition, segregation efficiency, transport and processing requirements, recovery yield, quality of secondary material or monomer, virgin-material displacement, and life-cycle impacts. These measurements would allow the field to progress from plausible circularity narratives toward comparable and decision-relevant evidence.

Accordingly, progress toward sustainable digital dentistry requires not only technically feasible recovery pathways but also quantitative evidence capable of linking material efficiency, recovered-material quality, safety, and environmental performance. The principal measurement gaps that currently prevent such evaluation are summarized in the following section.

### 3.6. Quantitative Evidence Gaps and Measurement Priorities

Despite increasing interest in dental PMMA recovery and circularity, the available evidence remains insufficient for quantitative comparison of waste-management and recycling scenarios. Three gaps are particularly relevant: the amount of PMMA waste generated per functional unit, the contamination characteristics that determine whether a waste stream is suitable for recovery, and the biological safety of recovered materials intended for reintegration into dental applications. These limitations restrict comparison between studies and prevent technical recyclability from being translated directly into validated clinical or environmental recommendations.

First, PMMA waste generation is rarely quantified using a standardized functional unit. Subtractive CAD/CAM manufacturing clearly produces unused disc remnants and milling residues [7,23,24,61], but the literature does not consistently report initial blank mass, mass incorporated into the finished device, milling residue mass, recoverable fraction, or material-utilization efficiency per restoration or prosthesis. General dental waste audits demonstrate that mass-based measurement is feasible [25], but differences in waste classification and reporting units limit comparison between studies. Future material-flow studies should therefore report PMMA input and output using device- or workflow-specific indicators, such as g of PMMA waste per device, percentage of blank utilization, percentage of source-segregated material recovered, and mass of virgin material displaced.

Second, contamination remains insufficiently quantified. Dental PMMA residues differ in their origin and potential exposure to processing fluids, polishing materials, other polymers, and, for clinically used devices, biological contaminants. Although feedstock purity and segregation are recognized as important determinants of recycling performance [27,28,38,39,40,41], the reviewed literature does not establish validated quantitative contamination thresholds for accepting or rejecting dental PMMA waste for mechanical recycling or depolymerization. Future studies should characterize contaminant type and concentration and determine their effects on recovery yield, MMA purity, secondary-material properties, and processing requirements. Such data are necessary before source-segregation categories can be converted into evidence-based acceptance criteria for recycling.

Third, biological evidence for recycled dental PMMA is available but remains limited. Studies using recycled denture-base PMMA provide selected in vitro mechanical, surface, and microbial-adherence outcomes [52,54], while evidence derived specifically from particles recovered from digitally milled PMMA discs remains focused primarily on material performance [23]. These findings support proof of concept but do not establish the biological safety of repeated clinical reintegration. Important unresolved endpoints include residual MMA and other leachables, cytotoxicity after aging and repeated recycling, particulate release, microbial behavior, and longer-term in vivo or clinical biocompatibility. Recovered PMMA or MMA intended to substitute virgin dental material should therefore be evaluated not only for mechanical equivalence but also for chemical purity and biological safety over clinically relevant aging conditions.

Finally, these material-flow, contamination, and safety indicators should be linked to recovery and life-cycle metrics. Recovery yield, energy and purification requirements, transport distance, rejected fractions, recovered-material quality, and effective substitution of virgin PMMA or MMA remain incompletely reported. Without these data, a high recovery yield cannot by itself demonstrate environmental benefit. Future studies should combine standardized material-flow measurements with explicit functional units and comparative life-cycle scenarios to determine whether prevention, reuse, mechanical recycling, depolymerization, or disposal provides the lowest overall burden under realistic dental-laboratory conditions [30,31,32,58,61]. The immediate research priority is therefore not to define universal recycling thresholds from the current evidence, but to generate the quantitative datasets required to establish and validate such thresholds.

## 4. PMMA Performance in Digital Dentistry

### 4.1. Performance Determinants

PMMA remains a principal prosthodontic polymer because it combines acceptable esthetics and biocompatibility with processability, repairability, affordability, and extensive clinical experience [4,5,63]. Improvements in formulation, curing, reinforcement, and industrial processing have reduced limitations associated with porosity, residual monomer, dimensional instability, and fracture [5,13,21]. Conventional heat- and auto-polymerized resins, modified formulations, industrial CAD/CAM blanks, and printable methacrylate systems should nevertheless be distinguished because their composition and processing histories affect both performance and recoverability [59,66].

Performance reflects the interaction between molecular architecture and processing. Molecular weight, chain entanglement, cross-linking, conversion, porosity, additives, and reinforcement influence strength, toughness, solvent resistance, and biological behavior [4,5]. Tacticity affects chain mobility and glass-transition behavior, but it does not by itself establish the clinical superiority or stereoregular composition of a commercial dental blank [83]. Cross-linking may improve dimensional and solvent stability while restricting direct melt reprocessing; similarly, fillers and impact modifiers may enhance first-use properties but complicate separation and purification.

Industrially polymerized CAD/CAM PMMA generally exhibits high conversion, low porosity, and lower residual MMA than auto-polymerized materials [5,6,64,84,85,86]. Comparative studies often report favorable mechanical, surface, and biological outcomes for milled PMMA [84,85,87], but superiority is not universal and depends on formulation, aging, specimen geometry, and test protocol [86]. The advantages are therefore manufacturing consistency, dimensional stability, low residual monomer, and biological tolerance rather than uniform superiority in every mechanical outcome [6,63,64,88].

### 4.2. Digital Manufacturing Routes

Prepolymerized discs provide standardized feedstock and support reproducible fabrication, while their mechanical performance is generally favorable compared with conventionally processed PMMA [6,64,85,89]. Stored designs may facilitate replacement and avoid repetition of selected clinical and laboratory stages. These benefits are environmentally relevant only when they reduce remakes, extend service life, or avoid other resource-intensive procedures.

Subtractive production also generates disc remnants, connectors, chips, and fine particles. Clean remnants from a known blank may be comparatively suitable for recovery, whereas coolant-contaminated particles, mixed dust, and clinically used devices are more difficult streams. Additive manufacturing may use material more selectively, but most printable dental resins are cross-linked methacrylate systems rather than thermoplastic PMMA. Common methacrylate chemistry does not imply equivalent performance or recyclability, and washing, post-curing, supports, unused resin, and long-term stability must be considered [7,22,90].

### 4.3. Clinical Function and Life-Cycle Performance

PMMA is used for complete and partial dentures, provisional restorations, implant-supported provisional prostheses, occlusal splints, surgical guides, diagnostic mock-ups, custom trays, orthodontic appliances, and maxillofacial prostheses [5,7,63,90,91,92,93] (Table 3). Digital files may simplify reproduction [7,10], but environmental performance also depends on longevity, repairability, remake frequency, manufacturing yield, and end-of-life handling. Alternative polymers and ceramics present different trade-offs involving cost, esthetics, processing, bonding, repair, and recovery [21,47,63,94]. Comparisons should therefore use an equivalent clinical function and service period rather than material mass or recyclability alone.

Clinical performance is therefore a life-cycle variable. A device that fails early or cannot be repaired may consume more material and clinical resources than an initially lower-impact alternative. Conversely, recyclability does not compensate for inadequate safety or durability. PMMA assessment should integrate conventional outcomes with service life, repairability, blank utilization, waste mass, segregation quality, recoverability, and retained material value. This link between function and material flow provides the basis for the following evaluation of environmental burdens and circularity.

## 5. Environmental Sustainability of PMMA

### 5.1. Life-Cycle Perspective and System Boundaries

The performance differences discussed in the preceding section have environmental consequences because accuracy, durability, repairability, and replacement frequency affect the resources required to deliver a clinically acceptable restoration. Sustainability is therefore not an intrinsic property of PMMA or of a particular manufacturing route. It is a system-level outcome that should be assessed for a defined clinical function across raw-material supply, production, transport, fabrication, use, maintenance, and end-of-life management [30,31,33,95].

**Figure 1:**
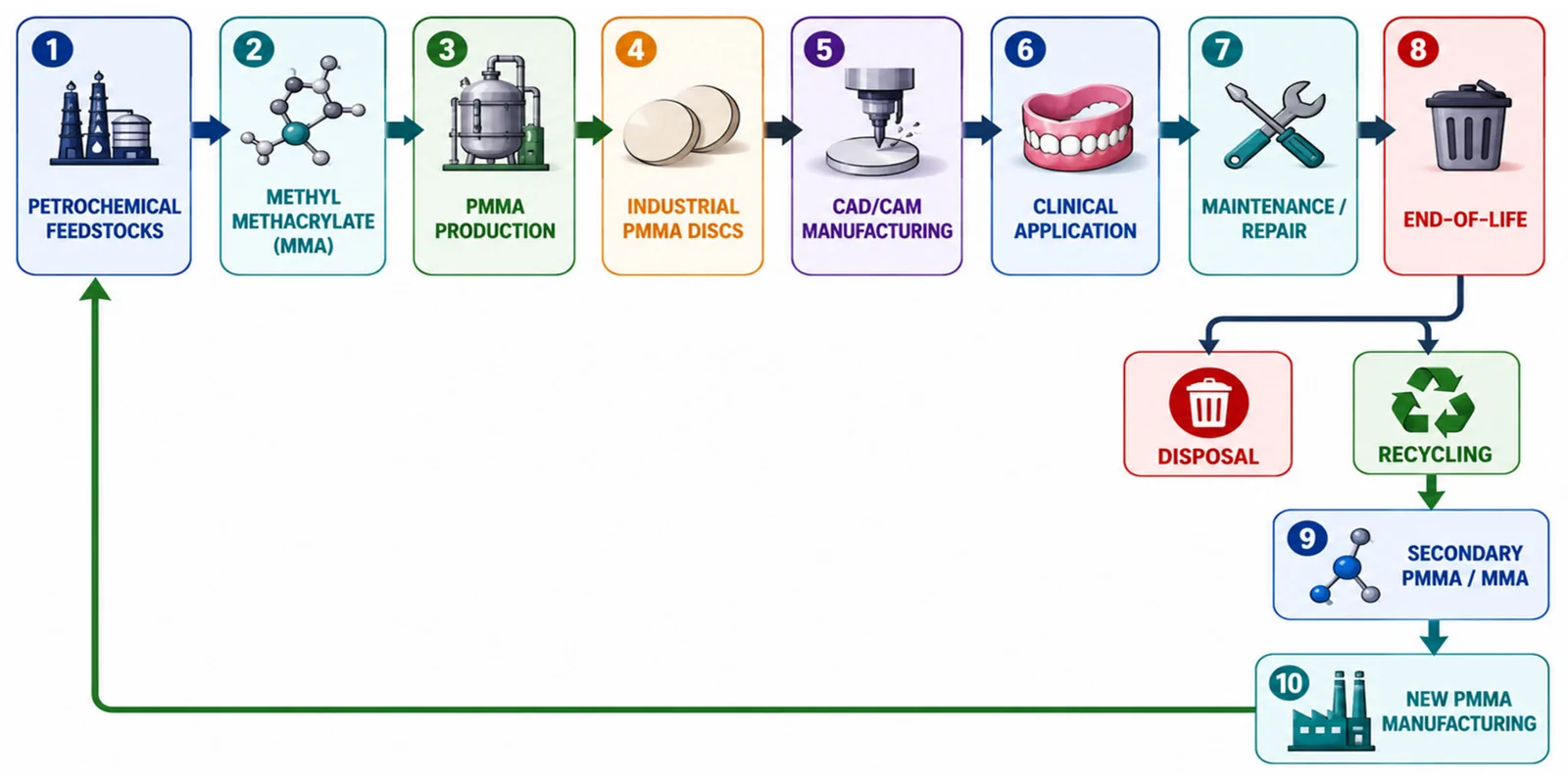
Life cycle of PMMA in Digital Dentistry. Arrows indicate the direction of material flow; the branching arrows represent disposal and recycling pathways, while the green return arrow indicates the circular recovery loop.

This distinction is important because PMMA is widely used in prosthodontics and small changes in material consumption or service life may accumulate across many treatments [51,95]. Its upstream burden begins with predominantly petrochemical feedstocks and the synthesis of MMA, followed by polymerization and industrial conversion into powders, resins, or prepolymerized blanks [36,40,96]. Dental processing then adds electricity demand, consumables, transport, and material losses. Figure 1 places these stages within a single analytical representation.

An LCA must consequently define the functional unit, system boundary, energy mix, transport assumptions, expected service life, and end-of-life scenario before alternatives are compared [30,31,97]. Comparing only the mass of material entering a machine, for example, can overlook remakes, post-processing, equipment use or differences in clinical longevity.

Future LCA studies of dental PMMA should define a functional unit that reflects the clinical or material function being compared. Depending on the research question, appropriate units may include one clinically acceptable PMMA device over a defined service life, a defined mass of PMMA waste managed, or a defined mass of recovered PMMA/MMA capable of substituting virgin material. Dental LCA studies demonstrate the use of clinically defined functional units, such as one dental restoration or one complete treatment procedure, to enable comparison between alternative materials or workflows [78,98]. For plastic recycling systems, methodological guidance distinguishes production-based functional units (e.g., a unit mass of recycled material produced) from waste-intake-based functional units (e.g., a unit mass of waste managed), because these address different comparative questions [99]. Accordingly, product-based and waste-management-based functional units should not be considered interchangeable in future LCA studies of dental PMMA circularity.

Scenario analysis should distinguish, where data permit, at least a virgin-material baseline, current waste-management practice, and one or more recovery scenarios. Recovery scenarios should explicitly report assumptions regarding collection efficiency, contamination or rejection rates, recovery yield, transport distance, energy source, purification requirements, recovered-material quality, and the amount of virgin PMMA or MMA effectively displaced. Sensitivity analyses should prioritize parameters likely to influence comparative results, particularly electricity mix, transport, recovery yield, device service life, and substitution assumptions.

### 5.2. Potential Benefits and Burdens of Digital Manufacturing

Digital workflows can improve standardization, accuracy, and reproducibility, while industrially polymerized CAD/CAM materials may support consistent clinical performance [6,64,89]. Digital records can also reduce physical model production in selected workflows, facilitate communication, and permit a restoration to be reproduced without repeating every preceding step [10,61,97]. These features may reduce remakes and replacement-related consumption, but only when the gains in quality and service life outweigh the burdens introduced by the digital system.

Those burdens differ by manufacturing route. Subtractive fabrication uses equipment, electricity, compressed air, cooling, tools, and prepolymerized discs, and it removes material that is not incorporated into the final device [15,89,97]. Additive manufacturing can improve geometric material utilization, yet it also requires resin handling, washing, post-curing, supports in some applications, and management of uncured or contaminated residues [7,22,90]. Consequently, neither route should be labeled environmentally preferable without a functionally equivalent comparison.

Alternative feedstocks and fillers represent further possibilities rather than demonstrated solutions. Bio-based routes to monomers and comparisons with polymers such as polylactic acid may reduce reliance on fossil carbon, whereas recycled zirconia and eggshell-derived hydroxyapatite have been investigated as waste-derived reinforcements [48,50,51,96]. Their net benefit nevertheless depends on processing energy, transport, formulation performance, service life, and compatibility with subsequent recovery.

### 5.3. Environmental Evidence and Remaining Uncertainties

The most visible downstream concerns are persistent waste and fine particles produced during milling, finishing, and polishing. PMMA-derived particles may enter workplace air or wastewater, but dental-specific data on release rates, environmental fate, and exposure remain limited [56,100,101]. These concerns warrant control and measurement, but the current evidence does not support precise estimates of their contribution to the total environmental burden.

A similar limitation applies to comparative LCA. Few studies provide dental PMMA-specific inventory data across the complete treatment pathway, and results are sensitive to geography, electricity generation, equipment utilization, transport, and assumed restoration life [30,31,61,97]. Polymer additives and incomplete recycling inventories introduce additional uncertainty [102]. Table 4 therefore distinguishes plausible effects from the evidence needed to verify them.

### 5.4. From Technical Recyclability to Environmental Performance

PMMA can be processed through mechanical recycling or chemically depolymerized to recover MMA, and continuing technical development has been reported for both pathways [23,27,28,39,40,41,42,52,60]. This technical potential is relevant to circular dentistry, but it should not be equated with an established closed loop for dental PMMA. Dental residues can be small, dispersed, mixed with other materials, or contaminated, and collection and traceability systems are not uniformly available.

Recycling produces an environmental benefit only when recovered material substitutes for virgin production and the avoided impacts exceed those of collection, transport, sorting, cleaning, and reprocessing [27,28,35,95,103,104]. Thus, recyclability, recycled content, recovery yield, and net life-cycle impact are related but non-interchangeable indicators. The next section examines the waste streams generated by digital dental workflows, because their quantity, form, purity, and location determine which recovery options are practically credible.

## 6. PMMA Waste Generation in Digital Dentistry

### 6.1. Origin of Waste in Digital Workflows

The preceding life-cycle analysis identifies waste characteristics as a practical constraint on PMMA circularity. Digital dentistry does not simply eliminate conventional waste: it changes its composition and location. Digital records can reduce impression and model materials in selected workflows, while CAD/CAM production and post-processing create new polymer-rich streams [62,95,97]. Their environmental relevance depends on the amount generated and on whether the material remains sufficiently clean and identifiable for recovery.

Subtractive manufacturing is an important and readily identifiable source of solid PMMA residues because restorations are produced by removing material from prepolymerized discs [23,62,95]. Waste arises as chips, connector remnants, unused disc segments, and fine particles. Additive workflows generate a different mixture that can include supports, failed prints, uncured resin, washing media, and post-processing residues; these streams should not be assumed to have the same handling or recycling requirements as solid milled PMMA.

### 6.2. Sources, Physical Form, and Recoverability

Subtractive milling of prefabricated PMMA blanks generates unused material and milling residues, whereas customized blocks have been proposed to reduce resin consumption and milling time [7,15,22]. The amount and physical form of the resulting waste are expected to depend on restoration geometry, blank dimensions, nesting strategy, and machine configuration; however, these relationships have not been consistently quantified. Finishing and polishing add dust and surface particles that are harder to capture than coarse remnants and may contribute to occupational or environmental exposure [100,105]. Clinical waste includes provisional restorations, fractured or replaced dentures, relined prostheses, guides, mock-ups, and expired materials.

Physical form, composition, and contamination are more informative than the generic label “PMMA waste”. Clean, source-separated offcuts and chips have greater recovery potential than mixed dust or devices exposed clinically [23,26,27]. In practice, however, polymer fractions are often discarded with mixed laboratory waste, so technically recoverable material loses value through inadequate segregation [62,106]. Table 5 classifies the main streams without implying that technical recyclability guarantees actual recovery.

### 6.3. Material Utilization and Quantitative Evidence

Material utilization is a key indicator for subtractive production, yet standardized reporting is uncommon. Most CAD/CAM studies prioritize accuracy, fit, or mechanical performance and do not report the mass of the initial blank, final device, collected residue, and unusable remainder. Consequently, comparisons between machines, designs, or nesting protocols remain uncertain. Customized blocks and alternative milling strategies have been proposed to reduce resin consumption and processing time [15]. At minimum, future studies should report material yield, waste per restoration, waste per disc, and the fraction collected separately.

The literature nevertheless provides several indicative values and proof-of-concept findings (Table 6). The only dental workflow-specific mass estimates identified in the reviewed literature were reported by Ong et al. [95] as illustrative values rather than as results of a standardized material-flow study. In the workflow described, a complete denture weighing approximately 12–14 g was associated with 120–150 g of unused disc material and 150–180 g of fine milling powder, whereas nesting 30 crowns within one disc generated approximately 55 g of waste [95]. These values illustrate the potential magnitude and workflow dependence of material losses but should not be interpreted as representative estimates for dental PMMA milling. Material yield should therefore be measured directly using clearly defined mass balances. Likewise, the high monomer-recovery yields reported under optimized depolymerization conditions do not account for the collection and sorting efficiency of heterogeneous dental waste streams.

Direct evidence specific to dental residues remains narrower (Table 7). Recycled CAD/CAM particles have been incorporated into heat-cured denture-base resin at 10 and 20 wt.%, with flexural performance maintained and surface hardness improved in the reported experiment [23]. Thermal and chemical routes can recover MMA at high yield, but optical quality, economics, and subsequent polymer performance may be affected by ageing, impurities, capital cost, and recovered-monomer value [28,39,41,107,108]. Clean segregation and reliable identification are therefore preconditions [26,43].

### 6.4. Environmental and Occupational Implications

Discarded PMMA embodies upstream burdens associated with feedstock production, MMA synthesis, polymerization, transport, and subsequent fabrication [26,27,28,29,96]. Inadequate segregation can also transfer polymer residues and fine particles to mixed waste, wastewater, or landfill pathways [109]. Milling, trimming, and polishing may generate airborne or waterborne micro- and nanoplastic particles; although dentistry-specific exposure and risk data remain limited, the issue justifies effective extraction, capture, and responsible disposal [100,101,105,110].

No internationally harmonized method currently defines how dental laboratories should measure PMMA waste or particle release. Laboratory investigations dominate the evidence, whereas real-world mass balances and exposure studies are scarce [100]. This gap limits LCA inventories and makes claims of waste reduction difficult to compare. Harmonized sampling, particle characterization, and clinically relevant exposure protocols should therefore accompany conventional manufacturing outcomes [100].

### 6.5. Management and Prevention Priorities

Current management remains predominantly linear. Although general dental guidance recommends segregation, recycling, and responsible disposal, dedicated systems for PMMA from digital workflows are not routinely implemented [111]. Common barriers include dispersed waste generation, uncertain contamination, absent collection infrastructure, weak economic incentives, and limited professional awareness [35,62,106,112]. Any proposed recovery route must also comply with local occupational, infection-control, transport, and waste regulations.

Prevention should precede recycling: improved nesting, fuller use of partly consumed blanks, design optimization, fewer failed jobs and remakes, and longer clinical service can reduce waste at source. For unavoidable residues, separation by formulation and contamination status, mass recording, and traceable collection can preserve recovery options [23,102]. Implementation requires coordination among clinicians, laboratories, manufacturers, collectors, and policymakers [112]. PMMA waste management thus forms the operational link between material performance and circular economy goals [33,95], providing the basis for the recycling technologies evaluated in the following section.

## 7. Recycling Pathways for Dental PMMA

### 7.1. Matching the Recovery Route to the Waste Stream

Dental PMMA waste is not a single feedstock: clean milling offcuts, fine dust, failed devices, and clinically exposed appliances differ in particle size, formulation, traceability, and contamination. A circular strategy must therefore match each stream to a technically and environmentally credible route rather than treat recyclability as a universal material property [26,33,82]. Research on zirconia milling residues illustrates the broader relevance of source-specific recovery within digital dentistry [57].

**Figure 2:**
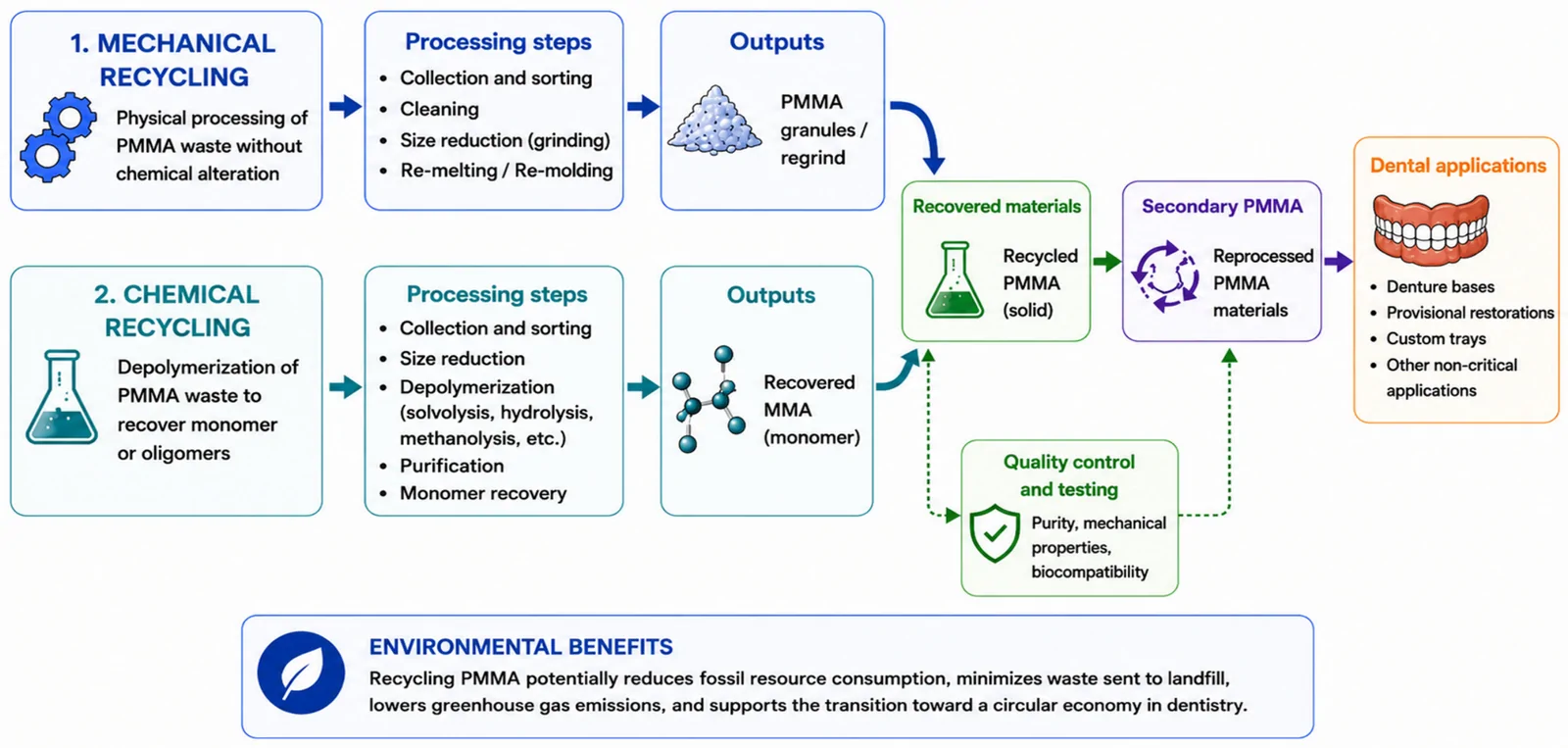
Overview of mechanical and chemical recovery routes for dental PMMA. Solid arrows indicate the principal processing and material-flow pathways toward secondary PMMA and potential dental applications, whereas dashed green arrows indicate the quality-control and testing steps required to verify purity, mechanical properties, and biocompatibility before reuse.

PMMA recovery can preserve the polymer through mechanical reprocessing, purify it through solvent-based approaches, or depolymerize it to methyl methacrylate (MMA) for subsequent polymerization [27,39,95]. Selection depends on feedstock purity, scale, required product quality, infrastructure, economics, and the impacts of collection and processing. Figure 2 summarizes these routes as options, not as evidence that a dental closed loop is already operating.

To maintain terminological consistency, related concepts are distinguished throughout this review in accordance with contemporary circular-economy terminology [113]. Recyclability refers to the technical potential of a material or product to undergo recycling, whereas recovery refers more broadly to processes through which material or resource value is retrieved from a waste stream. Circularity is treated as a system-level concept describing the extent to which resource value is retained or recirculated and should therefore not be inferred from recyclability alone. Sustainability is used as the broader concept encompassing environmental, social, and economic considerations. Accordingly, the term environmental benefit is reserved for situations in which a reduction in environmental burden is demonstrated relative to an explicitly defined comparator rather than inferred solely from recyclability or recovery.

### 7.2. Mechanical and Solvent-Based Recycling

Mechanical recycling generally comprises collection, sorting, cleaning, grinding, and incorporation of the secondary polymer into a new formulation [23,26]. It is most suitable for homogeneous, source-separated preclinical residues. Copolymers, additives, polishing media, cements, metals, other plastics, and biological contamination can reduce product consistency or prevent the route altogether [23,62,114]. For dental laboratories, clean chips and disc remnants are therefore more plausible feedstocks than captured dust or used appliances.

Repeated thermal and shear histories can lower molecular weight or alter performance; therefore, recycled content and the number of processing cycles require validation [115]. Industrial studies indicate that well-controlled PMMA streams may tolerate multiple cycles with limited deterioration, but these results cannot be transferred directly to clinical applications [116]. Dental evidence is currently limited chiefly to proof-of-concept incorporation of clean CAD/CAM particles in denture-base resin [23]. Solvent-based purification may retain polymer value and improve purity, but solvent recovery, residuals, safety, and limited adoption must be included in any assessment [73,117,118].

### 7.3. Chemical Recycling and MMA Recovery

Chemical recycling cleaves PMMA chains to recover MMA, which can be purified and repolymerized. Thermal, catalytic, hydrothermal, microwave-assisted, solvent-assisted, and reactive-extrusion approaches have been investigated [71,119,120,121,122]. Thermal depolymerization is the most mature route, but yield alone is insufficient: energy demand, emissions, purification, scale, feedstock quality, and the substitution of virgin MMA determine environmental performance [114,123].

Dental resin studies have demonstrated MMA recovery across laboratory, technical, pilot, and reactor-optimization scales [41,42,60,124]. Recovered monomer from virgin and aged PMMA has been repolymerized with comparable reported mechanical properties, although impurities from aged waste reduced optical transparency [108]. Chemical recycling may avoid the cumulative degradation associated with repeated mechanical processing [125], yet capital expenditure, plant utilization, recovered-monomer quality, and MMA market price remain decisive for economic viability [107].

Although standardized quantitative data on PMMA waste generation in dental laboratories remain scarce, experimental studies have reported process-level data for the depolymerization of dental PMMA residues. Table 8 summarizes the available quantitative outcomes from studies using dental PMMA waste, including process scale, feed mass, operating conditions, liquid-product yield, and MMA composition. These parameters are presented separately from material-flow indicators because recovery under controlled experimental conditions does not represent the overall proportion of PMMA waste that could be recovered in routine dental practice.

The available quantitative evidence demonstrates substantial process dependence. In the scale-up study by Ribeiro et al., liquid yield decreased from 95.63 wt.% at laboratory scale to 61.67 wt.% at technical scale and 59.18 wt.% at pilot scale, accompanied by a marked increase in the gaseous fraction [41]. Similarly, dos Santos et al. reported liquid yields between 48.20 and 55.50 wt.% under pilot-scale conditions, with MMA concentrations in the recovered liquid varying with temperature and reaction time [60]. These differences preclude the use of a single representative recovery efficiency and reinforce the distinction between demonstrated technical recoverability and real-world dental circularity.

Accordingly, mechanical and chemical routes are complementary. Mechanical processing is simpler for clean, local residues when the secondary product can tolerate some property variation; depolymerization can recover higher-value monomer but requires aggregated feedstock and industrial infrastructure. Table 9 compares the pathways without assigning clinical suitability to recycled output before mechanical, biological, and regulatory validation.

### 7.4. Evidence from International and Industrial Initiatives

This review did not identify a national protocol dedicated specifically to collecting, documenting, and recycling PMMA milling residues from dental laboratories. Existing practice is governed mainly by broader plastic, healthcare, hazardous-waste, or laboratory-waste frameworks [26,33,126]. This limited evidence should not be interpreted as proof that no local program exists; protocols may be regional, commercial, or unpublished.

The Finnish FilaPriDe project is directly relevant because it addresses acrylic waste from dental laboratories and reports substantial nightguard production losses and more than 500 kg of annual PMMA waste from two large laboratory companies [127]. At European industrial scale, Röhm’s alliance seeks to aggregate recyclable PMMA, while MMAtwo developed a value chain for scrap collection and MMA recovery [128,129,130]. These initiatives demonstrate organizational and technical capacity, but not yet a standardized route for routine dental waste.

Japanese work has combined LCA, material flow analysis, resource availability, and hazard evaluation in PMMA recycling-system design [131,132]. Chinese studies have advanced catalytic depolymerization and continuous reactive extrusion [120,133]. Frameworks in Sweden, Canada, Australia, and the United States encourage segregation and circular resource use, but the reviewed sources did not provide dental-PMMA-specific operational protocols [33,126]. More broadly, automotive, construction, electronics, and acrylic-sheet sectors show that aggregation and quality control can support PMMA recovery, although their scale and feedstock differ materially from dentistry [73,107,114,115,134].

### 7.5. A Provisional Laboratory-Level Protocol

Technical feasibility does not resolve the logistical barriers. Dental waste is dispersed among small generators; contamination and uncertain formulation complicate sorting; collection volumes may not cover transport and processing costs; and clinical reuse faces safety and regulatory requirements. Table 10 therefore proposes a conservative laboratory-level protocol. It is not a validated international standard and does not replace local infection-control, occupational, transport, or waste legislation.

Implementation would require manufacturers to disclose composition and support traceability, laboratories to segregate and measure material, collectors to aggregate compatible streams, and recyclers to report recovery yield and destination. The circular performance of PMMA should then be judged by collected mass, usable recovered output, virgin material substitution, safety, and net life-cycle impact, not by technical recyclability alone [114,116]. This systems perspective provides the operational basis for the sustainability assessment framework developed in Section 8.

## 8. Sustainability Assessment Framework for PMMA in Digital Dentistry

### 8.1. Purpose and Scope of the Framework

The preceding sections show why PMMA cannot be judged by a single attribute. Clinical durability may reduce replacement, efficient manufacturing may still generate substantial waste, and technical recyclability does not demonstrate collection, safe reuse, or environmental benefit. Assessment must therefore connect material performance, resource use, life-cycle impacts, circularity, clinical, and societal value [82,95,104,112,135].

Table 11 presents a conceptual decision support framework, not a validated score, certification method, or substitute for LCA. Its structure is informed by ISO 59004:2024, which establishes circular economy principles and terminology, and ISO 59020:2024, which provides a process for defining system boundaries and selecting circularity indicators [113,136]. Reviews of circularity metrics caution that indicators remain fragmented, often emphasize recycling, and may be weakly connected to environmental outcomes [135,137,138].

### 8.2. Interpretation Across the Five Dimensions

Material performance remains the entry condition: mechanical reliability, dimensional stability, esthetics, biocompatibility, and clinically relevant longevity determine whether a PMMA device is fit for purpose [5,63,64]. Manufacturing efficiency then records how much blank or resin, energy, tooling, and rework are required for that function. This dimension should include failed jobs and remakes rather than only nominal machine yield, particularly for subtractive workflows [23,62,109].

Environmental performance requires life-cycle methods and should cover feedstock, polymer production, fabrication, transport, use, maintenance, and end-of-life within an explicit functional unit and system boundary [30,31,97]. Circularity is narrower: it describes the preservation of material value through collection, traceability, reuse, mechanical reprocessing, or monomer recovery. It depends on infrastructure and implementation, not only polymer chemistry [32,33,135,136]. The two dimensions must remain distinct because a higher recycling rate does not necessarily produce a lower life-cycle burden.

Clinical and societal value provides the final constraint. Patient safety, treatment quality, economic feasibility, accessibility, occupational protection, and regulatory compliance determine whether an environmental strategy can be responsibly adopted [12,105,106]. The framework is intended to support transparent evaluation across all dimensions, without implying that strengths in one area can compensate for unacceptable shortcomings in another.

### 8.3. Application and Reporting Logic

Application of the framework should begin by defining the clinical function, comparator, system boundary, and assessment scenario. Indicators should then be selected for each of the five dimensions, with the corresponding data source, measurement unit, uncertainty, and evidence level reported transparently. Representative indicators include service life, material yield, energy consumption per acceptable device, waste generation, collected fraction, usable recovered output, virgin-material substitution, remake rate, cost, and regulatory compliance. Finally, improvements achieved at one stage of the life cycle should be evaluated for potential burden shifting elsewhere in the system [30,31,82].

**Figure 3:**
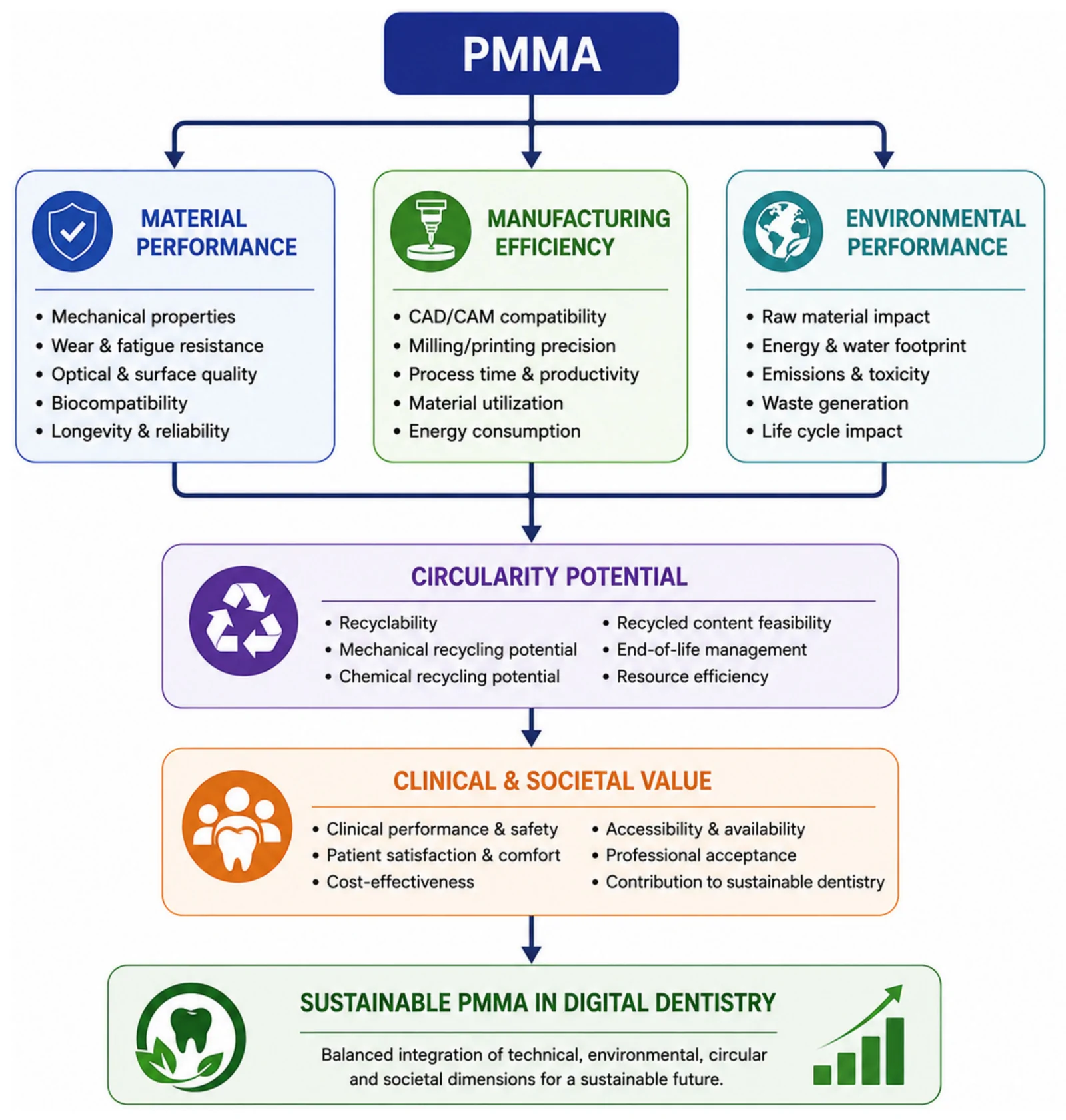
Integrated dimensions linking material performance, manufacturing efficiency, environmental performance, circularity potential, and clinical and societal value in the evaluation of PMMA for digital dentistry.

The framework can support comparisons of manufacturing routes, new formulations, laboratory improvement plans, recycling scenarios, and the design of future LCA studies. Operationalization will require PMMA-specific measurement protocols, reference values, and validation. In particular, circularity indicators should be coupled to life-cycle outcomes and should distinguish waste generated, waste collected, material recovered, and virgin feedstock actually displaced [103,135,136,137,138]. Figure 3 summarizes the relationships among the dimensions.

### 8.4. Research Priorities

Near-term research priorities include developing real-world material and energy inventories, establishing consistent functional units, generating clinical service-life data, measuring particle release, and validating recovery chains under practical conditions. Digital technologies may facilitate, but not guarantee, environmental improvements: AI could optimize material nesting, while digital twins may support process control and predictive maintenance [139,140]. Likewise, bio-based MMA, renewable energy, and advanced recovery technologies have the potential to reduce environmental burdens, but each requires comparative performance evaluation and life-cycle evidence before any environmental advantage can be substantiated [96,141].

The proposed framework should therefore be viewed as a structured research agenda derived from the available evidence rather than as a validated assessment tool. Future development should focus on refining indicators, engaging stakeholders, assessing inter-laboratory reproducibility, establishing weighting or decision rules where appropriate, and validating the framework against measured environmental and clinical outcomes. Maintaining a clear distinction between a conceptual framework and a validated assessment tool is essential for the interpretation and application of the proposed approach.

### 8.5. Economic, Regulatory, and Cross-Sector Implementation Barriers

The transition from technical PMMA recovery to routine implementation also depends on economic feasibility. Recycling costs extend beyond the recovery process itself and include segregation, collection, transport, preprocessing, purification, quality control, and management of rejected fractions. Industrial-scale analyses show that the economics of PMMA depolymerization are strongly influenced by feedstock quality, capital investment, recovery yield, and the value and quality of regenerated MMA [107]. These findings cannot be transferred directly to dental laboratories, where waste volumes are smaller, and collection and transport requirements differ. Accordingly, dental-specific assessments should compare the total cost of recovered PMMA or MMA with virgin-material and disposal costs while accounting for recovery yield and virgin-material displacement.

Regulatory requirements represent an additional barrier when recovered PMMA or MMA is intended for renewed clinical use. Technical recyclability alone does not establish suitability for incorporation into a dental medical device. Under the European medical-device framework, manufacturers must demonstrate conformity with applicable safety and performance requirements through risk management, clinical evaluation, technical documentation, and quality-management procedures [142]. Biological evaluation further requires assessment within a risk-management framework, while chemical characterization addresses material composition, process-related contaminants, extractables, leachables, and degradation products [143,144]. Therefore, clinical reintegration of recovered dental PMMA would require controlled material identity and composition, traceable processing, consistent performance, and appropriate chemical and biological safety evidence rather than technical recovery alone.

Implementation will also require collaboration among dental laboratories, material manufacturers, waste-management operators, specialized polymer recyclers, researchers, and regulatory bodies [81,82]. A pragmatic first step would be pilot collection of clean, source-separated manufacturing waste, particularly unused CAD/CAM remnants and identifiable milling residues, with simultaneous measurement of waste volume, contamination, recovery yield, cost, and environmental performance. Such partnerships could determine whether regional collection or manufacturer take-back systems are feasible and generate the evidence required before progression toward clinical-grade closed-loop recycling.

## 9. Discussion

### 9.1. Interpreting PMMA Performance Through a Life-Cycle Lens

The findings of this review indicate that clinical performance and environmental performance should not be considered independently. Strength, dimensional stability, biocompatibility, repairability, and service life influence failure, remake, and replacement rates and therefore the material and clinical resources required to deliver a functional restoration [5,6,15,84,85,86]. Although industrially polymerized CAD/CAM PMMA can provide greater material homogeneity, lower residual-monomer content, and reproducible manufacturing [15,84,86], these characteristics translate into environmental advantages only when they contribute to longer service life or reduced resource consumption. This interpretation is consistent with life-cycle and sustainable-healthcare approaches that assess biomaterials in relation to clinical function, resource use, environmental burdens, and end-of-life management [30,31,32,33,80], as well as recent syntheses emphasizing the integration of environmental considerations into responsible material selection in prosthodontics and dental plastics [67,95].

### 9.2. Digitalization Is a Conditional, Not Intrinsic, Environmental Benefit

Digital dentistry can improve precision, standardization, data transfer, and reproducibility while potentially reducing the use of impression materials, stone models, transport, and remakes [7,10,15,22,145]. However, these advantages do not establish an intrinsic environmental benefit, because burdens may shift toward industrially produced blanks and resins, electricity, equipment, data infrastructure, consumables, and post-processing [30,33,61,97]. This trade-off is particularly relevant to subtractive PMMA manufacturing, where accurate devices are produced from high-quality prepolymerized blanks but milling generates chips, connectors, and residual blank material [7,15,22,95]. Consequently, the environmental performance of digital PMMA workflows should be evaluated at the system level rather than inferred from digitalization alone [146].

### 9.3. Technical Recyclability Does Not Establish Dental Circularity

The reviewed evidence supports the technical recoverability of PMMA through mechanical reprocessing, solvent-based approaches, and depolymerization to MMA [23,27,39,41,42]. However, technical recyclability alone does not demonstrate a functioning circular system. Dental PMMA waste streams differ in formulation, particle size, traceability, and contamination, and their practical recovery therefore depends on source segregation, collection, safe handling, appropriate processing infrastructure, and quality requirements for recovered materials [32,41,76,77]. Direct dental studies provide proof of concept for selected recovery pathways, whereas evidence for routine closed-loop recycling, repeated clinical-grade reintegration, and displacement of virgin PMMA or MMA remains limited. Consequently, current evidence supports the technical potential for PMMA recovery more strongly than demonstrated circularity in routine dental practice [30,31,32,78].

### 9.4. Research and Implementation Priorities

The principal limitation of the current evidence base is the scarcity of real-world data on dental PMMA material flows, life-cycle impacts, collection, occupational exposure, and verified recycling performance [5,6,15,22,23,30,31,41]. Future studies should therefore prioritize standardized reporting of material inputs and outputs, waste generation, energy and consumable use, service life, collection and recovery yields, virgin-material displacement, and uncertainty, using explicit functional units and system boundaries [147]. Circularity indicators should be interpreted alongside environmental outcomes rather than as substitutes for them [135,136,138,148].

Emerging strategies—including AI-assisted design and nesting, digital twins, bio-based MMA, hybrid material systems, and advanced recovery technologies—may improve material efficiency or reduce dependence on fossil resources, but their benefits require comparative validation under clinically relevant and life-cycle conditions [51,96,114,139,140,141,149]. More broadly, eco-design should prioritize efficient material use, repairability, service-life extension, and recovery-compatible formulations, shifting attention from end-of-life treatment alone toward waste prevention and value retention [70,150,151].

## 10. Limitations

This critical narrative review was designed to integrate heterogeneous evidence across dental materials, digital manufacturing, LCA, and polymer recycling; it was not designed as a systematic review or meta-analysis. Searches of PubMed, Scilit, OpenAlex, and ScienceDirect were supplemented by Google Scholar and citation chasing, but the absence of subscription-based citation indexes, restriction to English-language sources, and the narrative search design may have resulted in missed studies. Coverage may be particularly incomplete for industrial reports, regional waste programs, non-indexed protocols, and polymer engineering literature. Consequently, statements that no dedicated national dental PMMA protocol was identified should be interpreted as a finding of this search, not proof that no local initiative exists.

The included evidence was methodologically heterogeneous. Studies used different materials, formulations, devices, processing conditions, comparators, and outcome measures. Much of the dental literature evaluates mechanical, biological, or manufacturing performance, whereas environmental studies frequently examine broader dental workflows or non-dental PMMA systems. This heterogeneity excluded quantitative synthesis and limits direct comparison among conventional, subtractive, and additive routes. Numerical estimates for blank utilization, waste generation, and monomer recovery are therefore context-dependent and should not be treated as universal values.

Environmental evidence specific to dental PMMA remains sparse. Few studies provide complete inventories, consistent functional units, real-world laboratory mass balances, clinical service life data, particle-release measurements, or verified recycling outcomes. Several interpretations consequently rely on indirect or transferable evidence from polymer science, industrial recycling, sustainable healthcare, and general dentistry. This review attempted to make this distinction explicit, but selection and interpretation of such evidence necessarily involve author judgment.

Finally, the laboratory waste-management protocol and sustainability framework are conceptual outputs of the evidence synthesis. They have not undergone stakeholder consensus, feasibility testing, inter-laboratory reproducibility assessment, weighting, regulatory review, or external validation against measured clinical and environmental outcomes. They should therefore be used to structure research and pilot implementation, not as validated standards, certification criteria, or clinical decision rules.

## 11. Conclusions

Current evidence supports the technical recoverability of selected dental PMMA waste streams, including controlled material reuse and MMA recovery through depolymerization. However, these findings should be interpreted primarily as proof-of-concept evidence rather than as demonstration of a validated closed-loop system for dental PMMA. Quantitative evidence remains limited regarding waste generation per functional dental unit, source-segregated collection, contamination, recovery under routine laboratory conditions, repeated material reintegration, and effective substitution of virgin PMMA or MMA. Similarly, environmental benefit cannot presently be inferred from technical recyclability or recovery alone and requires comparative life-cycle assessment using explicit functional units, system boundaries, and scenario assumptions.

Accordingly, the sustainability assessment framework and waste-management approach proposed in this review should be regarded as conceptual and hypothesis-generating rather than as validated clinical, environmental, or regulatory protocols. Their value lies in integrating material performance, manufacturing efficiency, waste generation, recovery, life-cycle assessment, and implementation requirements into a structured basis for future investigation. Prospective material-flow studies, standardized quantitative reporting, comparative LCA, assessment of recovered-material quality and safety, economic and regulatory evaluation, and real-world validation are required before these approaches can support evidence-based implementation in routine dental practice.

## Figures and Tables

**Table 1:** Structured search and evidence-selection approach.

Category	Description
Review design	Critical narrative review supported by a structured literature search
Bibliographic and discovery sources	PubMed, Scilit, OpenAlex, and ScienceDirect
Complementary searching	Targeted Google Scholar searches, backward and forward citation chasing, and searches of standards and official technical sources
Coverage	English-language literature published from January 2000 to June 2026
Core concepts	PMMA; poly(methyl methacrylate); dental; prosthodontics; CAD/CAM; digital manufacturing; milling waste; recycling; depolymerization; monomer recovery; LCA; circular economy
Evidence categories	Direct dental evidence; adjacent dental evidence; indirect polymer evidence; contextual evidence
Selection basis	Relevance to the review questions, methodological transparency, directness of evidence, and contribution to critical comparison or implementation analysis
Synthesis	Critical thematic synthesis; no meta-analysis or pooled effect estimates

**Table 2:** Characteristics, key quantitative/performance outcomes, and critical appraisal of the evidence included in this review.

Study	Design/Relationship to Review Question	Material or Process	Key Quantitative/Performance Data	Main Relevance/Limitation
Ivanova et al., 2026 [47]	Narrative review/contextual dental evidence	Contemporary dental polymers, including PMMA	≈116 publications synthesized; no PMMA-specific quantitative waste or recycling outcomes reported.	Provides contemporary dental-polymer context; does not directly assess PMMA recovery or circularity.
Ferreira et al., 2025 [42]	Experimental process study/direct dental-PMMA evidence	Semi-batch depolymerization of dental PMMA waste; laboratory, technical, and pilot scales	Depolymerization was evaluated from laboratory to pilot scale (up to 20 kg feed). At technical scale, 425 °C produced ≈81% liquid yield, compared with ≈61% at 450–475 °C; liquid fractions contained >90% MMA.	Strong direct evidence of scale-dependent MMA recovery; does not demonstrate collection feasibility, dental-grade repolymerization, or net LCA benefit.
Elzahar et al., 2022 [48]	In vitro material study/adjacent waste-valorization evidence	Recycled CAD/CAM zirconia nanoparticles incorporated into PMMA	Recycled ZrO_2_ incorporated at 0.01, 0.1, 0.3, and 0.5 wt.%; 0.3 wt.% provided the best overall mechanical performance; 0.5 wt.% reduced impact strength; no significant difference in cell viability at 24 h, although slight cytotoxicity was observed at 0.5%.	Demonstrates dental waste valorization, but the recycled phase is zirconia rather than PMMA.
Salim and Muhsin, 2020 [49]	In vitro material study/adjacent CAD/CAM waste-valorization evidence	Recycled PEEK milling waste used as filler in PMMA	Recycled PEEK particles (~150 μm) incorporated at 1 and 2 wt.%; 2 wt.% produced a significant increase in surface hardness and reduction in surface roughness versus unmodified PMMA.	Shows reuse of CAD/CAM polymer waste, but the recovered material is PEEK rather than PMMA; no recovery-yield or LCA data.
Yerou et al., 2026 [50]	Experimental composite study/adjacent bio-waste evidence	Eggshell-derived hydroxyapatite incorporated into dental PMMA	HAp incorporated at 10, 20, and 30 wt.%; 10 wt.% provided the best balance of tensile/compressive and thermal performance; higher loadings produced agglomeration. Tg increased relative to pure PMMA (~68 °C), reaching ~71–72 °C at 30 wt.% HAp.	Demonstrates bio-waste valorization and material substitution, not PMMA recovery.
Charasseangpaisarn et al., 2023 [51]	In vitro blend study/adjacent material-substitution evidence	PMMA/PLA blends	PMMA/PLA ratios of 100/0, 75/25, 50/50, 25/75, and 0/100 were tested. At selected processing temperatures, blend flexural strengths were approximately 76–78 MPa and comparable to neat PMMA; Tg decreased from 102.4 °C for PMMA to 80.8, 68.4, and 61.8 °C as PLA content increased.	Demonstrates partial substitution with a bio-based polymer; no recycling or comparative LCA was performed.
Krishnamoorthi et al., 2022 [52]	In vitro material study/direct dental-PMMA recycling evidence	Recycled denture-base PMMA incorporated into heat-cured PMMA	r-PMMA, 10–50 wt.%; Ra increased from 0.10 μm (control) to 0.11–0.16 μm, while Vickers hardness decreased from 20.35 VHN (control) to 16.67–13.84 VHN (10–50 wt.% r-PMMA). All groups met ISO 20795-1:2013 [53] polishability and translucency requirements.	Direct evidence for PMMA reuse, but increasing recycled content adversely affected surface properties; long-term safety and life-cycle effects were not evaluated.
Halib et al., 2025 [54]	In vitro material study/direct dental-PMMA recycling evidence	Heat-cured denture-base PMMA containing 20 and 50% recycled PMMA powder	Flexural strength remained high across recycled-PMMA groups; the 50% r-PMMA group showed 125.80 ± 17.53 MPa. No significant differences in hardness were reported among groups. *Candida albicans* adhesion was lowest in the 20% r-PMMA group (186.33 CFU).	Provides combined mechanical and microbial-adherence evidence for recycled dental PMMA. However, the recycled material originated from conventional denture-base PMMA rather than digital CAD/CAM waste, and biological evaluation was limited to microbial adhesion; cytotoxicity, residual monomer/leachables, aging, repeated recycling, and clinical safety were not assessed.
Hijazi et al., 2025 [23]	In vitro material study/direct dental-PMMA evidence	Fine (<400 μm) and whole particles recovered from milled PMMA discs; 10 and 20 wt.%	Fine r-PMMA, 10/20 wt.%: flexural strength 92.52/92.13 MPa versus 96.41 MPa control; hardness 18.32/18.73 VHN versus 17.67 VHN control. Ra remained 0.09–0.13 μm across groups. Whole particles reduced flexural strength and hardness; all flexural values remained >65 MPa.	Strongest direct mechanical-recycling evidence; particle size is critical. Aging, repeated recycling, biocompatibility, and clinical-scale implementation remain untested.
Rivera et al., 2025 [55]	Narrative/clinical review/contextual digital-dentistry evidence	Milled versus additively manufactured complete dentures	No primary quantitative PMMA waste or LCA data reported; milling is described as leaving unused blank material, whereas additive production may improve material utilization.	Useful workflow context, but environmental advantages are inferred rather than demonstrated quantitatively.
Yao et al., 2026 [56]	Experimental biological study/adjacent environmental-health evidence	Dental material microplastics, including PMMA	PMMA IC_50_ values were 202.9 μg/mL for hPDLCs, 224.9 μg/mL for HOK cells, and 213 μg/mL for THP-1-derived macrophages; 100 and 200 μg/mL were subsequently used as low/high exposure levels and produced dose-dependent cellular toxicity and inflammatory responses.	Provides quantitative hazard evidence relevant to particulate PMMA waste; does not evaluate recovery or environmental exposure thresholds.
Alqutaibi et al., 2025 [57]	Scoping review/analogous dental-manufacturing evidence	Recycling of CAD/CAM zirconia residues	26 studies included; 12 investigated residual blocks and 14 milling powder; 3 studies used recycled zirconia as PMMA fillers. Reported examples include recycled zirconia flexural strength of 680 MPa versus 800 MPa for commercial material.	Demonstrates recovery challenges and opportunities for another CAD/CAM waste stream; zirconia findings cannot be transferred directly to PMMA.
Rojas Varela et al., 2026 [58]	Comparative LCA/adjacent dental-LCA evidence	Conventional versus digital ceramic-crown fabrication	Functional unit: one clinically finished crown; cradle-to-grave analysis. Reported GWP was approximately 5.2 kg CO_2_-eq for a single-visit digital route, 10.8 kg CO_2_-eq for a multi-visit digital lithium-disilicate route, 13.1 kg CO_2_-eq for the zirconia-disc route, and 9.2 kg CO_2_-eq for the conventional route; patient transport was a major contributor.	Demonstrates that environmental performance depends on workflow and scenario assumptions; not PMMA-specific and does not evaluate recycling.
Monalisa et al., 2025 [59]	Narrative review/contextual additive-manufacturing evidence	Dental additive manufacturing and 3D printing	No PMMA-specific quantitative waste or LCA outcomes reported; this review describes reduced material waste, on-demand manufacturing, and possible use of recycled/bio-based polymers.	Broad contextual evidence; sustainability claims are largely indirect rather than based on comparative dental LCA.
dos Santos et al., 2022 [60]	Pilot-scale experimental process study/direct dental-PMMA evidence	Thermal depolymerization of dental PMMA residues in a 143 L reactor	Feed 14.6–15.0 kg. Liquid yields were 55.50, 48.73, and 48.20 wt.% at 345, 405, and 420 °C; gas yields were 31.69, 36.60, and 40.13 wt.%. MMA concentration ranged from 83.454–98.975 area%; >98% MMA was obtained at 30–80 min.	Strong quantitative proof of MMA recovery; does not demonstrate dental-grade repolymerization, repeated reuse, or environmental superiority.
Ribeiro et al., 2024 [41]	Multi-scale experimental study/direct dental-PMMA evidence	Depolymerization at laboratory (0.1 L), technical (2 L), and pilot (143 L) scales	Depolymerization was evaluated at laboratory (0.1 L), technical (2 L), and pilot (143 L) scales. At technical scale, 425 °C produced ≈81.6% liquid yield, compared with ≈61% at 450–475 °C; the liquid fraction contained >90% MMA. Scale-up increased thermal gradients and affected product yield and composition.	Demonstrates scale effects relevant to industrial translation; feedstock was controlled and collection/logistics and LCA were not assessed.
Martin et al., 2022 [25]	Observational waste audit/adjacent dental material-flow evidence	Single-use plastics generated during routine dental care	152 clinical observations; mean 21 SUP items and 354 g of SUP waste per procedure, including setup and cleanup.	Demonstrates a reproducible mass-based dental waste-audit methodology; waste was not PMMA/CAD-CAM specific.
Rosenblatt et al., 2026 [61]	Comparative LCA/adjacent denture-workflow evidence	Traditional alginate impression versus intraoral scanning for removable prosthetic fabrication	Traditional workflows: 77.29 kg CO_2_-eq (local laboratory) and 94.67 kg CO_2_-eq (global); digital workflows: 65.22 and 78.63 kg CO_2_-eq, respectively. Digital scenarios were ≈16–17% lower; reduced appointments lowered travel-related emissions by ≈20%.	Quantifies workflow-level environmental differences, but results are scenario-specific and do not isolate PMMA production, milling waste, or recycling.
Gussgard and Jokstad, 2025 [62]	Systematic review/adjacent oral-healthcare polymer-waste evidence	Polymer waste and nano-/microplastic pollution in dental care	30 studies: 16 waste audits, 8 clinical nano-/microplastic studies, and 6 landfill-related experiments. Reported polymer waste varied approximately 81–384 g per patient.	Provides quantitative dental polymer-waste context; evidence is heterogeneous and not PMMA-specific.

3D, three-dimensional; PMMA, poly(methyl methacrylate); r-PMMA, recycled poly(methyl methacrylate); MMA, methyl methacrylate; PEEK, polyetheretherketone; PLA, polylactic acid; HAp, hydroxyapatite; ZrO_2_, zirconium dioxide; CAD/CAM, computer-aided design/computer-aided manufacturing; LCA, life-cycle assessment; GWP, global warming potential; CO_2_-eq, carbon dioxide equivalent; Ra, arithmetic mean surface roughness; VHN, Vickers hardness number; CFU, colony-forming units; IC_50_, half-maximal inhibitory concentration; hPDLCs, human periodontal ligament cells; HOK, human oral keratinocytes; SUP, single-use plastics; Tg, glass transition temperature; THP-1, human monocytic leukemia cell line; wt.%, weight percentage. Quantitative data are reported only when directly available from the included source; where PMMA-specific quantitative outcomes were not reported, this is explicitly indicated.

**Table 3:** Clinical uses of PMMA and their principal life-cycle relevance.

Application Group	Typical Routes	Life-Cycle Relevance
Dentures and large provisional prostheses	Conventional processing; CAD/CAM milling	Longevity, repairability, and remake rate; large blanks may generate recoverable remnants
Crowns, bridges, and implant provisionals	Conventional processing; CAD/CAM milling	Accuracy and fracture resistance must be balanced against offcuts and relatively high replacement turnover
Occlusal splints and orthodontic devices	Conventional, milled, or printed routes	Chemically different material systems require route-specific recovery assessment
Surgical guides, mock-ups, and custom trays	Milled or printed routes	Short service life increases the importance of material efficiency and source segregation
Maxillofacial and customized devices	Conventional and digital routes	Durability and repair can avoid resource-intensive replacement; composition and contamination affect recovery

**Table 4:** Life-cycle interpretation of PMMA in digital dentistry.

Life-Cycle Stage	Potential Benefit	Potential Burden	Evidence Needed
Feedstock and polymer production	Industrial quality control	Fossil resources and process energy	Product specific inventory and energy source
Digital fabrication	Precision and fewer remakes	Equipment energy, consumables, and milling or resin waste	Equivalent workflow comparison
Clinical service	Durability and reproducibility	Repair or premature replacement	Clinical service life and remake rate
End of life	Mechanical or monomer recovery	Collection, contamination, transport, and processing	Recovery yield and displaced virgin material

**Table 5:** Major PMMA waste streams in digital dental workflows.

Source	Typical Waste	Recovery Considerations
CAD/CAM milling	Coarse chips and fine particles	High potential if composition is known and collection is separate
Disc use and connector removal	Offcuts, remnants, and partly used blanks	Relatively clean; geometry may permit reuse before recycling
Finishing and polishing	Acrylic dust	Capture is difficult; mixtures and small particle size reduce recovery quality
Failed production	Mis-milled or failed devices	Potentially recoverable before clinical use if composition is traceable
Clinical replacement	Provisional restorations and denture bases	Contamination, additives, and mixed components require assessment
Expired material	Unused blanks or resins	Composition may be known, but route depends on condition and local rules

**Table 6:** Reported quantitative and qualitative indicators relevant to PMMA material efficiency and recovery.

Indicator	Reported Evidence or Interpretation	References
Material utilization in complete-denture milling	A final complete denture weighing approximately 12–14 g was obtained from the illustrated PMMA-disc workflow	[95]
Unused disc material	Approximately 120–150 g remained as unused disc material after milling one complete denture	[95]
Fine milling powder	Approximately 150–180 g of fine powder was generated during the illustrated complete-denture milling workflow	[95]
Waste from optimized crown nesting	Milling 30 crowns from one PMMA disc generated approximately 55 g of waste	[95]
Mechanical recycling	Feasible for clean, ground PMMA; properties depend on waste quality and formulation	[26,27]
Chemical depolymerization	Optimized processes commonly report approximately 90–98% MMA recovery under controlled conditions	[27,28]
Primary workflow hotspot	Subtractive milling is a clearly identifiable source of solid PMMA residues, although its relative contribution has not been quantified across dental workflows	[23,95]

**Table 7:** Direct evidence relevant to PMMA waste recovery.

Finding	Critical Interpretation	References
MMA recovery above 90 wt.%	Demonstrated under optimized depolymerization conditions; not a dental collection rate	[28,39,41]
Repolymerization of recovered MMA	Mechanical properties can be retained, while aged feedstock impurities may reduce transparency	[108]
Economic feasibility	Sensitive to capital expenditure and the market value of regenerated MMA	[107]
Dental residue incorporation	CAD/CAM particles tested at 10 and 20 wt.% in heat-cured PMMA with acceptable reported performance	[23]
Source segregation	Uncontaminated, traceable PMMA is required for credible mechanical or chemical recovery	[26]

**Table 8:** Quantitative outcomes reported for depolymerization and MMA recovery from dental PMMA waste.

Study	Dental PMMA Feedstock	Process Scale	Feed Mass/Reactor Volume	Process Conditions	Liquid Yield (wt.%)	Gas Yield (wt.%)	MMA in Liquid Product	Main Interpretation
dos Santos et al., 2022 [60]	Cross-linked PMMA-based dental resin scraps	Pilot	14.6 kg/143 L	345 °C, atmospheric pressure	55.50	31.69	83.454–98.975% area *	Lower temperature favored liquid recovery; MMA purity > 98% area was achieved during selected reaction intervals
dos Santos et al., 2022 [60]	Cross-linked PMMA-based dental resin scraps	Pilot	15.0 kg/143 L	405 °C, atmospheric pressure	48.73	36.60	83.454–98.975% area *	Increasing temperature reduced liquid yield and increased gas formation
dos Santos et al., 2022 [60]	Cross-linked PMMA-based dental resin scraps	Pilot	15.0 kg/143 L	420 °C, atmospheric pressure	48.20	40.13	83.454–98.975% area *	Higher temperature further increased gas formation; recovery performance remained strongly process-dependent
Ribeiro et al., 2024 [41]	PMMA-based dental resin scraps	Laboratory	40 g/0.1 L	Final temperature 450 °C; 60 min	95.63	0.73	Scale- and time-dependent	Very high liquid yield was obtained at laboratory scale
Ribeiro et al., 2024 [41]	PMMA-based dental resin scraps	Technical	625 g/2 L	Final temperature 455 °C; 100 min	61.67	35.90	Scale- and time-dependent	Scale-up substantially reduced liquid yield and increased gas formation
Ribeiro et al., 2024 [41]	PMMA-based dental resin scraps	Pilot	20 kg/143 L	Final temperature 458 °C; 130 min	59.18	32.32	Scale- and time-dependent	Pilot-scale performance confirmed technical feasibility but showed important scale-related heat-transfer limitations

* MMA values reported by dos Santos et al. [60] were obtained by gas chromatography–mass spectrometry (GC-MS) and reported as chromatographic area percentage; they should not be interpreted as mass recovery of MMA from the original PMMA feedstock.

**Table 9:** Comparison of PMMA recycling pathways.

Pathway	Recovered Output	Main Advantage	Principal Limitation	Current Relevance to Dental Waste
Mechanical	Secondary PMMA	Relatively simple; preserves polymer	Contamination and cumulative degradation	Most plausible for clean, segregated chips; dental evidence limited
Solvent-based	Purified polymer	Potentially high polymer purity	Solvent management and limited adoption	Potential route requiring process and safety validation
Thermal/chemical	Recovered MMA	Potential virgin-feedstock substitution	Energy, purification, cost, and industrial scale	Technically demonstrated; collection chain not established
Catalytic and emerging	MMA or purified products	Potential gains in selectivity or efficiency	Technology maturity and scale-up	Research and industrial development rather than routine practice

**Table 10:** Proposed protocol for managing PMMA milling waste in dental laboratories.

Step	Proposed Action	Purpose	Supporting Basis
1	Segregate PMMA at the source	Preserve clean material before mixing occurs	[26,127,128]
2	Separate chips, offcuts, dust, failed devices, and clinical waste	Distinguish different contamination and recovery profiles	[23,127]
3	Use labeled, closed, dry containers	Limit cross-contamination and retain traceability	[127,128]
4	Exclude gypsum, metals, saliva, cements, polishing media, and other polymers	Protect feedstock quality and worker safety	[26,128]
5	Record material identity, source, mass, and period	Enable mass balance, benchmarking, and future LCA	[129,131,132]
6	Prioritize reuse of suitable unused blank geometry, then mechanical recovery	Prevent waste before lower-value processing	[23,128]
7	Transfer eligible fractions to authorized depolymerization where available	Enable MMA recovery at an appropriate industrial scale	[41,42,120,129,133]
8	Do not return recycled material to clinical use without validation	Protect biological safety, performance, and regulatory compliance	[23]
9	Include waste indicators in laboratory quality management	Link material flow to accountability and improvement	[33,131,132]
10	Report utilization, waste, collection, and verified recycling rates separately	Avoid conflating waste collected with material actually recycled	[41,129,131,132]

**Table 11:** Conceptual sustainability assessment framework for dental PMMA.

Dimension	Decision Question	Representative Indicators	Interpretive Precaution
Material performance	Does the device safely deliver its clinical function?	Strength, wear, stability, biocompatibility, service life	Environmental gains cannot offset inadequate safety or function
Manufacturing efficiency	How effectively are resources converted into an acceptable device?	Material yield, energy, consumables, failed jobs, remakes	Compare functionally equivalent workflows
Environmental performance	What burdens occur across the defined life cycle?	Climate impact, energy, resources, transport, waste, particles	Use explicit functional unit, boundary, geography, and scenario
Circularity	How much material value is demonstrably retained?	Collection, traceability, recovered yield, recycled content, substitution	Do not equate recyclability or collection with verified recycling
Clinical and societal value	Is the option feasible, safe, accessible, and compliant?	Patient safety, occupational exposure, cost, access, regulation	Identify trade-offs and affected stakeholders

## Data Availability

No new data were created or analyzed in this study. Data sharing is not applicable to this article.

## Supplementary Materials

The following supporting information can be downloaded at: https://www.mdpi.com/article/10.3390/polym18172071/s1, Table S1: Complete database search strategies and numbers of records retrieved; Figure S1: Literature-identification and study-selection flow.

## Abbreviations

The following abbreviations are used in this manuscript:

3D	Three-Dimensional
AI	Artificial Intelligence
CAD	Computer-Aided Design
CAD/CAM	Computer-Aided Design/Computer-Aided Manufacturing
CFU	Colony-Forming Units
CO_2_-eq	Carbon Dioxide Equivalent
GC-MS	Gas Chromatography–Mass Spectrometry
GWP	Global Warming Potential
HAp	Hydroxyapatite
HOK	Human Oral Keratinocytes
hPDLCs	Human Periodontal Ligament Cells
IC_50_	Half-Maximal Inhibitory Concentration
ISO	International Organization for Standardization
LCA	Life-Cycle Assessment
MMA	Methyl Methacrylate
PEEK	Polyetheretherketone
PLA	Poly(lactic acid)
PMMA	Poly(methyl methacrylate)
PRISMA	Preferred Reporting Items for Systematic Reviews and Meta-Analyses
Ra	Arithmetic Mean Surface Roughness
SUP	Single-Use Plastics
Tg	Glass Transition Temperature
THP-1	Human Monocytic Leukemia Cell Line
VHN	Vickers Hardness Number
ZrO_2_	Zirconium Dioxide
