# Supplementary material for: Life-Cycle Performance of Poly(methyl methacrylate) in Digital Dentistry: A Critical Review of Material Efficiency, Waste Generation, and Circularity
Source: Polymers (Basel). 2026 Aug 26;18(17):2071. doi: 10.3390/polym18172071 (PMC13568213; doi:10.3390/polym18172071)
Supplement: Supplementary file 1 [file polymers-18-02071-s001.zip › polymers-4522350-Table S1.pdf]

Table S1. Complete database search strategies and numbers of records retrieved.

Database	Search fields and complete search strategy	Records retrieved
PubMed	(“PMMA” OR “polymethyl methacrylate”) AND (“digital dentistry” OR “CAD/CAM” OR milling OR “3D printing” OR “additive manufacturing” OR denture* OR prosthodontics OR “provisional restoration*” OR “interim restoration*”) AND (recycling OR sustainability OR “circular economy” OR waste OR depolymerization OR “environmental impact” OR microplastics)	650
Scilit	The PubMed conceptual strategy was adapted to the Boolean operators and search fields available in Scilit: (“PMMA” OR “polymethyl methacrylate”) AND (“digital dentistry” OR “CAD/CAM” OR milling OR “3D printing” OR “additive manufacturing” OR denture* OR prosthodontics OR “provisional restoration*” OR “interim restoration*”) AND (recycling OR sustainability OR “circular economy” OR waste OR depolymerization OR “environmental impact” OR microplastics). Because year-specific filtering was required, the search was performed separately for each publication year and the results were subsequently combined.	21
OpenAlex	Title field: PMMA OR “polymethyl methacrylate”; dental/manufacturing concept group: “digital dentistry” OR “CAD/CAM” OR milling OR “3D printing” OR “additive manufacturing” OR denture OR prosthodontics OR “provisional restoration” OR “interim restoration”; sustainability concept group: recycling OR sustainability OR “circular economy” OR waste OR depolymerization OR “environmental impact” OR microplastics. The three concept groups were combined using AND; terms within each group were combined using OR.	287
ScienceDirect—Search 1	Find articles with these terms: PMMA OR “polymethyl methacrylate”; title, abstract, or author-specified keywords: (“digital dentistry” OR “CAD/CAM” OR milling OR denture) AND (recycling OR sustainability OR waste)	3
ScienceDirect—Search 2	Find articles with these terms: PMMA OR “polymethyl methacrylate”; title, abstract, or author-specified keywords: (“3D printing” OR “additive manufacturing” OR prosthodontics OR “provisional restoration” OR “interim restoration”) AND (recycling OR sustainability OR waste)	1
ScienceDirect—Search 3	Title: PMMA OR “polymethyl methacrylate”; title, abstract, or author-specified keywords: (dental OR dentistry OR denture) AND (depolymerization OR “circular economy” OR “environmental impact” OR microplastics)	1

Note: The retrieval counts reported in Table S1 refer to the structured database searches. Three additional eligible studies were identified through complementary searching, including targeted Google Scholar searches and citation chasing, and were therefore not included in the database retrieval counts.
